# Joint analysis of the intention to vaccinate and to use contact tracing app during the COVID-19 pandemic

**DOI:** 10.1038/s41598-021-04765-9

**Published:** 2022-01-17

**Authors:** Marta Caserotti, Paolo Girardi, Alessandra Tasso, Enrico Rubaltelli, Lorella Lotto, Teresa Gavaruzzi

**Affiliations:** 1 Department of Developmental Psychology and Socialization, University of Padova, Padua, Italy; 2 Department of Statistical Sciences, University of Padova, Padua, Italy; 3 Department of Humanities, University of Ferrara, Ferrara, Italy

**Keywords:** Psychology, Diseases, Medical research

## Abstract

Pharmacological and non-pharmacological measures will overlap for a period after the onset of the pandemic, playing a strong role in virus containment. We explored which factors influence the likelihood to adopt two different preventive measures against the COVID-19 pandemic. An online snowball sampling (May–June 2020) collected a total of 448 questionnaires in Italy. A Bayesian bivariate Gaussian regression model jointly investigated the willingness to get vaccinated against COVID-19 and to download the national contact tracing app. A mixed-effects cumulative logistic model explored which factors affected the motivation to adopt one of the two preventive measures. Despite both COVID-19 vaccines and tracing apps being indispensable tools to contain the spread of SARS-CoV-2, our results suggest that adherence to the vaccine or to the national contact tracing app is not predicted by the same factors. Therefore, public communication on these measures needs to take in consideration not only the perceived risk associated with COVID-19, but also the trust people place in politics and science, their concerns and doubts about vaccinations, and their employment status. Further, the results suggest that the motivation to comply with these measurements was predominantly to protect others rather than self-protection.

## Introduction

Almost any country in the world has been hit by the SARS-CoV-2 pandemic. Since the first wave of the pandemic, when the development of safe and effective vaccines was still far away, the Italian government imposed several strict measures on the population, including lockdowns and protective behaviors such as physical distancing and wearing masks, that were quickly joined by the national digital Contact Tracing App Immuni (henceforth, CTA Immuni), suggested as a tool to limit the spread of COVID-19 by tracing and notifying at-risk users^1^. By March 12, 2021, the four main vaccines against COVID-19 have been approved for administration in the European Union^2^ and Italy^3^ and the roll out with specific categories of professionals and citizens has begun. Nonetheless, until adequate coverage is achieved^4^, the adoption of protective behaviors, including digital tracing of positive cases, would help to control the spread of SARS-CoV-2^5^. Indeed, vaccines were approved through emergency procedures, and some aspects are still unclear, as evidence is emerging. It is still unclear to what extent vaccinated people will pass the infection to others^6^ or how long will the protection last. Furthermore, while in the early stages of vaccination several population groups had to wait a considerable time before receiving the injection, making it more complicated to achieve coverage^7^, others, such as children younger than 5 years of age, as of December 2021, are still waiting for the results of the dedicated clinical trial^8^. Therefore, the two measures, pharmacological and non-pharmacological, are destined to coexist for some time to come. Additionally, it is unclear to what extent the available vaccines will protect from current and future variants of the virus^2^. Hence, it is crucial to investigate what common and specific factors characterize the acceptance of the two measures: vaccines and contact tracing apps. Knowing these factors could be of great help for policymakers and communicators, in both the immediate and long term. Given the importance that vaccine uptake and the use of CTAs have in the resolution of a pandemic, this study aims to investigate which factors affect the willingness to get vaccinated against COVID-19 and the intention to download the CTA Immuni. Although there might be numerous variables that moderate acceptance of protective measures, it is essential to investigate them more closely, to be able to promptly intervene in case of potential future medical emergencies.

CTAs register users’ proximity to notify people who recently interacted with anyone who was later diagnosed with COVID-19^9^. At the time of writing (September 2021), a total of 84 CTAs has been documented by the Ada Lovelace Institute^10^. In Italy, the CTA Immuni was developed by the Special Commissioner for the COVID-19 emergency and released in June 2020. The app works by keeping track of contacts between CTA Immuni users. In the case of a positive test, the user, in collaboration with an authenticated healthcare professional, is required to report the positivity for SARS-CoV-2 to the app. Subsequently, the app notifies the at-risk users, depending on the duration and proximity of the contact. In case of notification, the app recommends to at-risk users what to do, suggesting self-isolation (to minimize the spread of the virus) and contacting their family doctor (to receive the most appropriate care and to reduce the likelihood of severe complications).

As with other preventive measures, the effectiveness of CTAs depends on public adherence^9^, as they need high levels of continued usage to provide useful data and to work properly^11^. Despite governments encouraging the use of CTAs, adherence has been very low worldwide. For example, in countries like the United States, Switzerland, France, and Italy, the download of CTAs has been remarkably low^12,13^. Specifically, in Italy, 1 month after release, the CTA Immuni was downloaded by only 2.7 million people^14^ reaching 15.4 million people (25.5% of the national population) at the time of writing (September 2021)^15^.

Several studies investigated what psychological determinants impact people’s intention to download and use CTAs. For example, privacy and data concerns negatively affect the willingness to download CTAs^16–18^, as does the perception that CTAs are not effective or necessary to prevent the pandemic^19^. On the contrary, self-efficacy and general trust in app providers^16^, social influence and innovativeness^9^, attitudes toward technology^18^, trust in the state and apps perceived effectiveness^17^, besides utility^20^ and trust in the government^21^ are positively associated to the acceptance of CTAs. Attitudes towards CTAs are positively influenced by fears associated with the pandemic, perceived and personal threat, and lack of control^17,18,21–23^.

Similarly, it has been shown that participants who perceived COVID-19 as a very risky disease^24,25^ or who perceived their likelihood of becoming ill with COVID-19 as high^26^ showed higher vaccination intentions. Indeed, doubts about vaccinations in general reduced the intention to get vaccinated against COVID-19. Moreover, concerns about the safety of vaccines, production rates, and places of origin^27^, as well as doubts in general or about their efficacy, strongly reduce the intention to vaccinate against COVID-19^25^. If, on the one hand, vaccine adherence is greatly reduced by adherence to conspiracy theories^28^, on the other hand, the idea of getting vaccinated to protect loved ones, such as family members and close friends, positively influenced vaccination adherence^29^. Given this framework, we think it is important to determine whether people who have concerns about vaccination are more or less likely to download CTAs as, for example, some people might think that a CTA provides some protection from the virus. Whereas several studies investigated separately people’s willingness to download and use a CTA or to get vaccinated against COVID-19, no study, as far as we know, investigated how the two measures interact. We believe that a potential interaction deserves attention as the willingness to get vaccinated and using a CTA both allow us to protect ourselves from contagion. This is important as it has been shown that acceptance and adoption of very different protective behaviors are predicted by risk perception, and trust in politics and science^30^. Given that experts continue to emphasize that the next pandemic is a matter of “when, not if”^31^, the study of the characteristics that encourage, or hint, acceptance of the two measures, pharmacological and non-pharmacological, is of great importance in the communication and development of policies for the containment of uncertain and risky situations. Some predictors are likely to affect the acceptance of both measures, such as perceived risk or perceived threat^17,18,21–26^, trust in governments and science^17,21,28,32,33^ and a sense of personal responsibility towards others and the community, which could promote not only the willingness to get vaccinated^29^ but also downloading CTAs. At the same time, the two protective measures are very different and are likely to be affected by distinct predictors, for instance worries about safety and side effects might be specific for vaccines only^27^ and privacy might be a specific concern about CTAs^16,17^.

The aim of the present study is to jointly investigate the determinants of vaccine acceptance and willingness to download a CTA, combining the two strands of literature in a novel way. We investigate if previous vaccine behavior and having general doubts about vaccinations affects the intention to get vaccinated (H1), and if COVID-19 related perceived risk and trust in politics and science are factors influencing both protective measures (H2). Further, we investigated differences in willingness to adhere to the two protective measures to primarily protect oneself or others (H3). Specifically, we hypothesized:

### H1.a

High doubts about vaccinations should reduce the intention to get vaccinated against COVID-19.

### H1.b

Previous vaccine behaviors should increase the intention to get vaccinated against COVID-19.

### H2.a

High-risk perception related to COVID-19 should predict acceptance of both preventive measures.

### H2.b

High trust in politics and science should predict acceptance of both preventive measures.

### H3

People who report high intention to get vaccinated against COVID-19 and download CTA Immuni should do this mostly for others, rather than themselves.

In the present study, we performed our analyses on a dataset that was used for a prior publication^25^ of the research group, but focusing on different variables that will help answer the research questions presented. Specifically, we focused on some of the variables considered in the last of the three waves examined in our previous work, paying attention to the comparison between attitudes towards COVID-19 vaccines and the national CTA. To sum up, the present work aimed at exploring whether people show similar or specific attitudes towards the two protective measures, vaccine and CTA Immuni, and whether and how different attitudes towards these measures are affected by different determinants.

## Methods

### Participants

Data were collected online in Italy by sharing the study link on various social channels related to the research team, during the initial phase of the reopening after the first lockdown in Italy (May 11th–June 28th 2020). Participation was voluntary. Out of 668 participants who started the questionnaire, 6 were dropped because they did not provide informed consent and 214 because they did not complete all measures (Supplementary Table S1). Thus, in the final sample we considered 448 participants (70.8% female, age mean ± SD = 33.8 ± 13.9 years). All procedures performed in the study were in accordance with the ethical standards of the institution and with the 1964 Helsinki Declaration and its later amendments or comparable ethical standards. The project was approved by the ethical committee for Psychological Research of the University of Padova (Italy), with protocol number 3596/2020. Informed consent was obtained for all the participants.

### Procedure

The questionnaire investigated participants’ intention to download the national CTA Immuni and to get a vaccine against COVID-19 (if a vaccine were available, as the study was conducted in mid 2020). In both cases, we asked participants to answer on a numeric scale ranging from 0 (*not at all likely*) to 100 (*very likely*), calculating the level of hesitancy by the complement to 100. Immediately after, participants were also asked to indicate to what extent their intention to get vaccinated against COVID-19 or download the CTA Immuni, presented in randomized order, was meant to protect themselves and to protect others (0 = *not at all* to 4 = *very much*). Furthermore, we assessed risk perception associated with COVID-19 through three questions^25^ that investigated the perceived likelihood of being infected (0 = *not at all likely* to 100 = *extremely likely*), the perceived severity of the disease (0 = *not at all severe* to 100 = *extremely severe*), and the scare felt (0 = *not at all scared* to 100 = *extremely scared*). Subsequently, participants self-reported whether they had received the flu vaccine in the previous season (2019–2020) and how doubtful they were about vaccines in general (0 = *not at all doubtful* to 100 = *extremely doubtful*). The responses to this latter question were categorized into four categories, the first to keep into account the zero inflation (no doubts) and other categories based on the terciles of the remaining empirical distribution (low, medium, and high doubts). Participants were asked to report how much they believed their behaviors could help solve the COVID-19-related health emergency (0 = *not at all* to 100 = *absolutely*), and how much they trusted international, national, regional institutions, and scientific committees (0 = *not at all trusty* to 100 = *absolutely trusty*). Participants were also invited to answer a single Likert 7-items scale to measure people’s general tendency to believe in conspiracy theories^34^. Finally, participants reported a series of demographic information: gender, age, income, highest level of education, municipality of residence, and postal code. In addition, we asked for details on the type of employment contract of participants (employed, business owner, retired or unemployed and student). This last question was introduced because one of the features of the CTA Immuni is to notify users of potential at-risk contacts by inviting them to comply with a period of self-isolation. We believe that this aspect could have a differentiated effect according to the job category: indeed, if a condition of quarantine or preventive isolation implies a more or less significant economic loss for business owners, this loss does not exist for the other categories. The full questionnaire is available in the Supplementary Note. Data is openly available at https://osf.io/kqf4h/?view_only=35091d750d104a8c9781822d8b9d2cbf.

### Statistical analysis

#### Descriptive analysis

Data were summarized in frequency tables and figures (frequency for categorical variables, median and InterQuartile Range (IQR) for continuous variables), histograms, and boxplots. Non parametric tests (Wilcoxon and Kruskal–Wallis test) were computed to compare the distribution across strata given the predominant non-normal distribution of the continuous variables. Categorical variables were compared using chi-squared or Fisher’s exact test where expected frequencies in any combination were less than 10. Statistical significance was assumed at the 5% level. Statistical analysis was performed using R^35^ using packages ggplot2^36^ for the graphical representation.

#### COVID-19 perceived risk and trust in politics and science—exploratory factor analysis

Two different explorative factorial analyses were performed: the first one on the respondents’ scores of the likelihood of being infected, severity, and scariness for COVID-19, the second one on the trust in international institutions, national institutions, and scientific committees. Because these groups of variables were closely related (Supplementary Fig. S1), we wanted to extract only one index for each dimension. Specifically, factor analyses were based on the empirical variance–covariance matrix and in each analysis, a single factor was estimated (Supplementary Table S2) which represented the overall COVID-19 perceived risk and the trust in politics and science, respectively. The amount of variance explained by the one-factor solution was satisfactory (60.0% for COVID-19 perceived risk and 71.4% for trust in politics and science). The factor loadings for COVID-19 reported a high relevance of the scariness and severity with less importance of the likelihood of being infected, while for trust in politics and science all the three variables were highly relevant, in particular trust in scientific committees. The estimated factor scores were categorized in terciles (1st tercile = low risk; 2nd tercile = medium risk; 3rd tercile = high risk).

#### Bayesian bivariate Gaussian regression model

**Figure 1:**
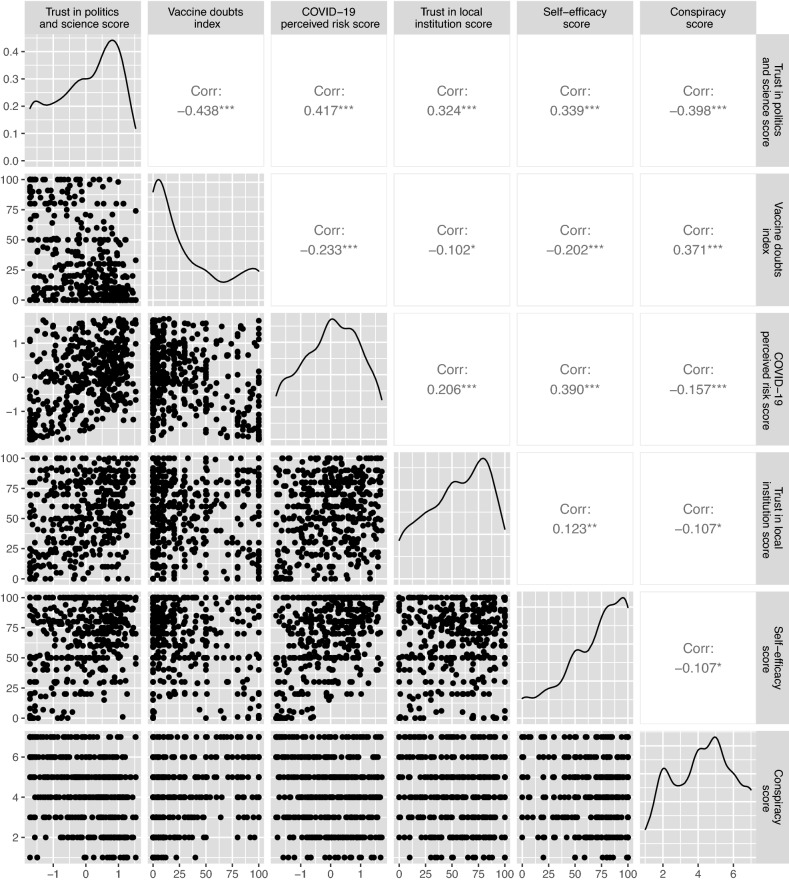
Marginal distribution and pairwise Spearman’s correlation between Trust in politics and science, Vaccine doubts index, COVID-19 perceived risk, Trust in local Institutions, Self-efficacy score and Conspiracy. Significance test is reported. Spearman’s rank correlation rho test: *p < 0.05, **p < 0.01, ***p < 0.001.

The willingness to get the COVID-19 vaccine and to download the CTA Immuni were highly correlated (Fig. 1). We employed a Bayesian Bivariate Gaussian (BBG) regression model, which is an extension of the standard linear regression model for the two correlated continuous response variables. The proposed estimation process at first performed an estimation of the coefficients on the basis of the maximized likelihood (ML) and then a Markov Chain Monte Carlo (MCMC) sampling starting from the ML estimates and with non-informative priors^37^. Coefficient estimates and the relative 95% Credible Interval (95% CrI) were obtained by the mean and the 2.5–97.5% percentiles of the a-posteriori distribution. We denote with Y = (Y_1,_Y_2_) the bivariate continuous variable associated with the likelihood to download CTA Immuni and to get the COVID-19 vaccine, respectively. Let ***X*** = [1, X_1_, X_2_,…, X_p_]^T^ be the vector of covariates, which is (p + 1)-dimensional. Then the BBG regression model is expressed as follows:

$$Y\sim {N}_{2}\left({\varvec{\mu}},\Sigma \right),$$

where ${\varvec{\mu}}=\left({\mu }_{1},{\mu }_{2}\right)$ and each position parameter ${\mu }_{j}={{\varvec{\beta}}}_{{\varvec{j}}}{\varvec{X}}$, with j = 1 or 2. The vector ${{\varvec{\beta}}}_{{\varvec{j}}}$ is the vector of parameters relative to the marginal response ${Y}_{j}$. The variance–covariance matrix $\Sigma$ is decomposed into $\Sigma =D\Omega D$ with

$$D=\left(\begin{array}{cc}{\sigma }_{1}& 0\\ 0& {\sigma }_{2}\end{array}\right)\, \text{and}\,\Omega = \left(\begin{array}{cc}1& \rho \\ \rho & 1\end{array}\right),$$

where ${\sigma }_{1}$ and ${\sigma }_{2}$ are two scale parameters and $\rho$ is the correlation parameter. In order to ensure the identifiability of the parameters (${\sigma }_{1}$, ${\sigma }_{2}$ > 0 and $\rho \in (\text{0,1}))$ the link functions were ${\eta }_{{\sigma }_{j}}=log\left({\sigma }_{j}\right)$ and ${\eta }_{\rho }=rhogit\left(\rho \right)$, with the function $rhogit\left(\rho \right)=\frac{\rho }{\sqrt{1-{\rho }^{2}}}$.

In order to understand which variables also influenced the scale and correlation parameter we extended the regression to the previous mentioned parameters as following

$${\eta }_{{\sigma }_{j}}={{\varvec{\gamma}}}_{{\varvec{j}}}{\varvec{X}},\boldsymbol{ }\text{and }{\eta }_{\rho }= {{\varvec{\beta}}}_{3}{\varvec{X}},$$

where ${{\varvec{\gamma}}}_{{\varvec{j}}}$ and ${{\varvec{\beta}}}_{3}$ are vectors of coefficient relative to the effect of the covariate related to the scales and correlation parameters. The effect of age was considered with a penalized smoothing splines basis (thin plate regression spline); we choose a number of 5 bases minimizing the Deviance Information Criterion (DIC) index. For simplicity and as a measure of comparison, we considered the same set of covariates for all the parameters, with a forward selection criterion considering the DIC index (Supplementary Table S3). The a-posteriori coefficients distribution was obtained after MCMC 10.000 iterations with a burn-in of 1000 iterations. The BBG regression model was estimated by R^35^ and the bamlss package^38^.

#### Cumulative logistic model

For both the measures, pharmacological and non-pharmacological, participants were asked to evaluate through two different questions to what extent they would adhere to each behavior in order to protect themselves or to protect others (1 = *not at all* to 4 = *very*). The reported values were summarized in the Supplementary Table S4. All the four sets of responses are highly and positively correlated with COVID-19 perceived risk. Considering that each participant expressed four responses (2 type of preventive measure × 2 altruistic sensitivity modality) thus the dataset was reshaped in a long format so that for each subject a single column included all the motivation score responses (on a 4-item Likert scale) as the dependent variable and the type of measure and altruistic sensitivity formed other two columns of covariates. To evaluate which factors influenced the motivation to take a measure against COVID-19, we employed a mixed-effects cumulative logistic model as follows

$$logit(P({Y}_{i}\le k))={\theta }_{k}-{(X}^{T}\beta +{\varepsilon }_{i}),$$

where k = 1,…,4 are the possible values and ${Y}_{i}$ is the observed value for the i-th observation; the parameters ${\theta }_{k}$ is the intercept which depends on k, while *β* is the vector of coefficients of the regression matrix X; ${\varepsilon }_{i}$ is the regression error at zero mean and constant variance that follows a normal distribution $N(0,{\sigma }_{\varepsilon }^{2}).$

Considering a hierarchical structure, given by the repeated responses for each subject, now we denoted with the response j for the i-th subject, the model takes the following form:

$$logit(P({Y}_{ij}\le k))={\theta }_{k}-{(X}^{T}\beta +{{\gamma }_{j}+\varepsilon }_{ij}),$$

where *β* are the coefficients of the fixed effect; the random effect parameter ${\gamma }_{j}$ follows a normal distribution $N(0,{\sigma }_{\gamma }^{2}$), while the residual error takes the form of ${\varepsilon }_{ij}\sim N(0,{\sigma }_{\varepsilon }^{2}).$ The regressors included in the matrix X were chosen by minimizing the Akaike Information Criterion (AIC) index testing the inclusion of the type of measure (COVID-19 vaccine or CTA Immuni), altruistic sensitivity (for myself, for the others), other covariates (e.g. age, gender), and individual susceptibility (the subject), this latter included as a random intercept.

In order to understand which factors influenced the belief in conspiracy theories, which was reported in a 7-point Likert scale, a second cumulative logistic regression model (without a random intercept) was fitted with the previous selection criteria. The results were presented using Odds Ratios (ORs) and 95% Confidence Interval (95% CI) by exponentiating the estimated coefficients. The model was estimated by R^35^ and the *ordinal* and *clmm* package^39^.

## Results

### Descriptive analysis

**Figure 2:**
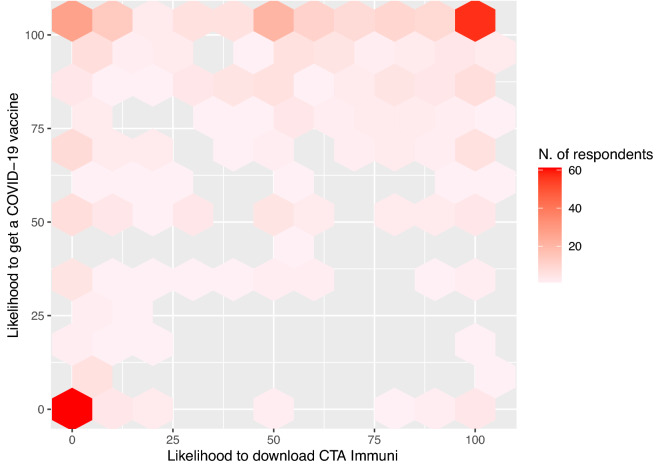
Joint distribution of the likelihood to get a COVID-19 vaccine and to download CTA Immuni.

Participants were mainly female (70.8%), the < 25 years age-class was the most represented, and they reported an educational level equally distributed between the high school or lower and the university degree or higher. Table 1 shows key participant characteristics by the degree of doubtfulness toward vaccines in general. Specifically, the adult age-class reported more doubts about vaccinations (p < 0.001) as well as those with the lower educational level (p = 0.004). A limited fraction of respondents reported business-owner employment (n = 53, 11.8%); this percentage increased among those who had many doubts about vaccinations (n = 25, 24.3%, p < 0.001). Only 11.2% of participants had the flu vaccine in the season 2019–2020, clearly associated with fewer vaccine doubts with respect to who did not take the vaccine (p = 0.006). The willingness to get a COVID-19 vaccine was greater than that to download the CTA Immuni (median score 90 vs. 50, Wilcoxon test p < 0.001), but both intentions decreased as vaccine doubts increased (both p < 0.001). Trust in politics and science was higher among participants with no or few vaccine doubts (p < 0.001), but not considering the trust in local institutions. The self-efficacy score was negatively correlated with the vaccine doubts (p < 0.001), while the score showed an opposite trend with the highest values among those reporting high vaccine doubts (p < 0.001). COVID-19 risk perception was high among all the categories of the vaccine doubts with the exception of that with the highest doubts (p < 0.001). Trust in politics and science positively correlated with that in local institutions and self-efficacy scores (Fig. 1, Spearman’s correlation; 0.32 and 0.34, respectively). A good correlation was reported between the score of COVID-19 perceived risk and the self-efficacy score (Spearman’s corr.: 0.39) and between vaccine doubts and conspiracy (Spearman’s corr.: 0.37). The likelihood to get the COVID-19 vaccine was highly correlated with the likelihood to download CTA Immuni (Fig. 2, Spearman’s corr = 0.43, rho test p < 0.001).

**Table 1:** Main characteristics of the participants, overall and by degree of vaccine doubts index.

Characteristics	Overall N = 448	Vaccine doubts index				p-value^1^
		No doubts N = 98	Low doubts N = 113	Medium doubts N = 134	High doubts N = 103	
Gender (females), N (%)	317 (71%)	68 (69%)	78 (69%)	94 (70%)	77 (75%)	0.78
Age, median (IQR)	27 (23, 46)	25 (22, 30)	25 (23, 37)	26 (22, 42)	44 (31, 50)	** < 0.001**
**Educational level, N (%)**						** < 0.001**
Middle school	33 (7.4%)	4 (4.1%)	2 (1.8%)	12 (9.0%)	15 (15%)	
High school	197 (44%)	48 (49%)	40 (35%)	62 (46%)	47 (45%)	
University degree or higher	218 (49%)	46 (47%)	71 (63%)	60 (45%)	41 (40%)	
**Family status, N (%)**						** < 0.001**
Single	220 (49%)	58 (59%)	69 (61%)	67 (50%)	26 (25%)	
Married—living together	186 (42%)	33 (34%)	34 (30%)	51 (38%)	68 (66%)	
Others	42 (9.4%)	7 (7.1%)	10 (8.8%)	16 (12%)	9 (8.7%)	
**Job, N (%)**						** < 0.001**
Employee	186 (42%)	34 (35%)	43 (38%)	53 (40%)	56 (54%)	
Business-owner	53 (12%)	7 (7.1%)	7 (6.2%)	14 (10%)	25 (24%)	
Retired-unemployed	35 (7.8%)	9 (9.2%)	8 (7.1%)	10 (7.5%)	8 (7.8%)	
Student	174 (39%)	48 (49%)	55 (49%)	57 (43%)	14 (14%)	
**Salary, N (%)**						0.084
< 15 k	23 (27%)	34 (35%)	32 (28%)	30 (22%)	27 (26%)	
15–55 k	99 (44%)	33 (34%)	45 (40%)	71 (53%)	50 (49%)	
> 55 k	44 (9.8%)	13 (13%)	12 (11%)	7 (5.2%)	12 (12%)	
Unknown	82 (18%)	18 (18%)	24 (21%)	26 (19%)	14 (14%)	
Flu vaccine in 2019–2020 done, N (%)	50 (11%)	16 (16%)	16 (14%)	16 (12%)	2 (1.9%)	
Likelihood to get a COVID-19 vaccine, median (IQR)	90 (50, 100)	100 (95, 100)	100 (87, 100)	80 (50, 100)	0 (0, 51)	** < 0.001**
Likelihood to download CTA Immuni, median (IQR)	50 (0, 88)	65 (30, 100)	55 (12, 94)	40 (3, 80)	0 (0, 50)	** < 0.001**
Trust in politics and science score, median (IQR)	0.12 (− 0.74, 0.82)	0.72 (− 0.12, 1.06)	0.56 (− 0.14, 1.00)	0.12 (− 0.54, 0.63)	− 0.89 (− 1.62, − 0.14)	** < 0.001**
Trust in local institution score, median (IQR)	60 (33, 80)	70 (35, 84)	55 (40, 80)	54 (38, 72)	60 (21, 80)	0.17
Self-efficacy score, median (IQR)	76 (50, 91)	80 (64, 100)	80 (66, 91)	70 (50, 85)	60 (30, 92)	** < 0.001**
Conspiracy score, median (IQR)	4 (3, 6)	3.5 (2, 5)	4 (2, 5)	4 (3, 5)	6 (5, 7)	** < 0.001**
COVID-19 perceived risk score, median (IQR)	0.04 (− 0.74, 0.75)	0.13 (− 0.72, 0.82)	0.15 (− 0.18, 0.88)	0.19 (− 0.28, 0.79)	− 0.89 (− 1.55, 0.34)	** < 0.001**
Direct COVID-19 contact, N (%)	248 (55%)	46 (47%)	61 (54%)	79 (59%)	62 (60%)	0.21

Tests are performed between characteristics and categories of vaccine doubts index.

Significance values are given in bold.

^1^Pearson’s Chi-squared test; Kruskal–Wallis rank sum test.

### Bayesian bivariate Gaussian model

The results of the estimated BBG regression model showed that vaccine doubts index exerted a progressive and greater adverse effect on the intention to get the COVID-19 vaccine (from low doubts: − 2.39; 95% CrI − 6.09–1.58, to high doubts − 38.42 points; 95% CrI − 46.39 to − 30.64) than on the intention to download CTA Immuni (from low doubts: − 7.43; 95% CrI − 17.64–2.67, to high doubts: − 14.60 points; 95% CrI − 25.26 to − 3.90; Table 2). Having received the flu vaccine in the season 2019–2020 increased the intention to get the COVID-19 vaccine and download CTA Immuni by 7.85 (95% CrI 4.24–11.66) and 11.30 (95% CrI − 1.28–23.37) points, respectively. The COVID-19 perceived risk showed a moderate effect in increasing the likelihood to get a COVID-19 vaccine (high perceived risk: + 12.73 points; 95% CrI 7.63–18.04) and a strong effect on the intention to download the CTA Immuni (high perceived risk: + 18.64 points; 95% CrI 9.68–27.88). Trust in politics and science greatly increased the intention to download the CTA Immuni, especially for those who most trusted politics and science (+ 29.52 points; 95% CrI 20.63–38.60). Gender did not affect the response variables. Business-owners job reported a lower intention to get the COVID-19 vaccine − 3.05 points; 95% CrI − 22.46 to − 4.21) as compared to employees, while a positive effect on CTA downloading was observed among retirees or unemployed (+ 14.58 points; 95% CrI 2.68–27.23). Educational level reported an effect only on the intention to get the COVID-19 vaccine: an increment of more than 14 points emerged among respondents with higher education with respect to those with lower schooling.

**Table 2:** Adjusted coefficients and 95% CrI estimated by a Bayesian bivariate Gaussian regression model for the position parameter (${\mu }_{1}$ and ${\mu }_{2}$) the score of likelihood to get a COVID-19 vaccine and to download CTA Immuni, and their correlation rhogit($\rho$) respect to the reference category.

	COVID-19 vaccine			CTA Immuni			rhogit($\rho$)		
	$\widehat{{\beta }_{1}}$	95% CrI		$\widehat{{\beta }_{2}}$	95% CrI		$\widehat{{\beta }_{3}}$	95% CrI	
Intercept	**57.44**	**44.36**	**70.77**	**30.00**	**13.96**	**46.82**	− 0.09	− 0.65	0.41
**Vaccine doubts index**									
Low (1, 14)	− 2.39	− 6.09	1.58	− 7.43	− 17.64	2.67	0.11	− 0.25	0.44
Medium (14, 50)	** − 11.76**	** − 16.42**	** − 7.33**	− 8.90	− 18.88	0.99	0.35	− 0.01	0.70
High (50, 100)	** − 38.42**	** − 46.39**	** − 30.64**	** − 14.60**	** − 25.16**	** − 3.90**	0.34	− 0.03	0.71
Flu vaccine 2019–2020 done [Yes]	**7.85**	**4.24**	**11.66**	11.30	− 1.28	23.37	0.03	− 0.34	0.41
**COVID-19 perceived risk score**									
Medium (− 0.431, 0.545)	**9.23**	**4.06**	**14.08**	2.40	− 5.34	10.27	− 0.16	− 0.43	0.12
High (− 0.545, 1.71)	**12.73**	**7.63**	**18.04**	**18.64**	**9.68**	**27.88**	** − 0.34**	** − 0.64**	** − 0.03**
**Trust in politics and science score**									
Medium (− 0.407, 0.623)	**8.29**	**2.77**	**14.27**	**15.40**	**6.31**	**23.98**	** − 0.41**	** − 0.68**	** − 0.11**
High (0.623, 1.53)	**11.44**	**5.57**	**17.63**	**29.52**	**20.63**	**38.60**	0.01	− 0.31	0.31
Gender (male)	1.63	− 2.27	5.34	1.19	− 4.85	7.68	− 0.20	− 0.47	0.02
**Job category**									
Business-owner	− **13.05**	− **22.46**	− **4.21**	− 3.98	− 12.88	5.36	**0.65**	**0.25**	**1.08**
Retired or unemployed	2.87	− 6.61	12.56	**14.58**	**2.68**	**27.23**	0.17	− 0.29	0.66
Student	− 1.30	− 5.62	3.30	− 2.10	− 11.20	7.55	0.20	− 0.11	0.52
**Education level**									
High school	**14.24**	**3.12**	**25.80**	− 1.88	− 14.94	10.57	0.20	− 0.27	0.69
University degree or higher	**15.08**	**2.85**	**26.81**	4.45	− 9.65	17.10	0.32	− 0.15	0.85

In bold 95% CrI outside the null effect.

**Figure 3:**
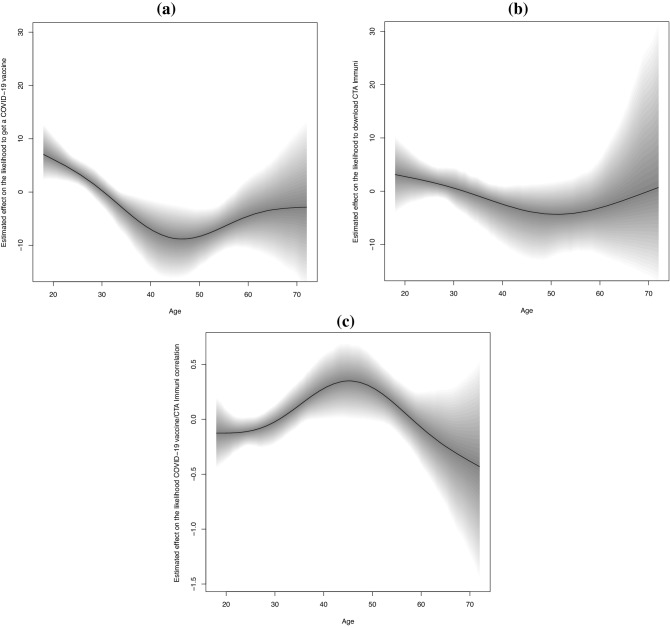
Adjusted effect of age estimated by a Bayesian Bivariate Gaussian regression model for the position parameter (${\mu }_{1}$ and ${\mu }_{2}$) of the score of likelihood to get a COVID-19 vaccine (**a**) and to download CTA Immuni (**b**), and their correlation (rhogit($\rho$), (**c**)).

Adjusted also by age with penalized cubic splines with 5 equally spaced knots (Fig. 3).

Reference category: Vaccine doubts index (No doubt), Flu vaccine 2019–2020 done (No), COVID-19 perceived risk score (Low), Trust in politics and science score (Low), Gender (Female), Job (Employee), Education level (Middle school).

The age-class did not significantly affect the likelihood to install the CTA Immuni, while adults aged 40–50 reduced intention to get vaccinated for COVID-19 (Fig. 3a,b). Concordance between the two measures increased between respondents with 40–50 years (Fig. 3c). A high perception of COVID-19 risk decreased CTA Immuni/COVID-19 vaccine concordance (rhogit($\rho$): − 0.34; 95% CrI: − 0.64 to − 0.03) compared with a lower risk perception, as did a medium trust in politics and science (rhogit($\rho$): − 0.41; 95% CrI: − 0.68 to − 0.11) compared with low trust. Also, being a business owner strongly increased the concordance between the adoption of these measures (rhogit($\rho$): 0.65; 95% CrI 0.25–1.08) compared to being an employee.

The variability of the intention to get the COVID-19 vaccine increased with vaccine doubts index while decreasing if the flu vaccine 2019–2020 was done, at the increasing of trust in politics and science, and in the highest educational level category. Variability in the intention to download the CTA Immuni increased with increasing trust in politics and science (Supplementary Table S5) and age (Supplementary Fig. S2).

The sample/theoretical quantile–quantile diagram of the a-posteriori regression residual error reported a discrete fitting to the normal distribution of the model (Supplementary Fig. S1), with a limited presence of departure from the normality on the low residuals tail both for the likelihood to get a COVID-19 vaccine and intention to download the CTA Immuni. However, given the non-bell shape of the response variables, the model provides an acceptable fit (Supplementary Fig. S3).

### Cumulative logistic models

Results of the mixed-effects cumulative logistic model are shown in Table 3: the intention to take a measure against COVID-19 was lower among those who declare to do it for themselves rather than for others (OR 0.71; 95% CI 0.58–0.88), while the motivation of getting vaccinated for COVID-19 was three times higher than to download the CTA Immuni. A negative effect on the motivation was reported by age (1-year increase OR 0.98; 95% CI 0.96–0.99) and by having conspiracy beliefs (6–7 vs < 5 score point, OR 0.50, 95% CI 0.29–0.85). Trust in politics and science and COVID-19 perceived risk highly increased the importance given to the preventive measures.

**Table 3:** Adjusted ORs estimated by a mixed-effects cumulative logistic regression model of the motivation to take a measure against COVID-19.

	OR	95% CI	p-value
Intercept 1|2	0.03	(0.02–0.05)	** < 0.001**
Intercept 2|3	0.12	(0.08–0.19)	** < 0.001**
Intercept 3|4	0.78	(0.50–1.21)	0.268
**Measure motivation**			
For others (reference)	1.00	–	–
For myself	0.71	(0.58–0.88)	
**Measure type**			
CTA Immuni (reference)	1.00	–	–
COVID-19 vaccine	3.21	(2.58–3.99)	** < 0.001**
**Gender**			
Females (reference)	1.00	–	–
Males	0.78	(0.44–1.39)	0.398
Age (+ 1 year increase)	0.98	0.96–0.99	**0.009**
**Educational level, N (%)**			
Middle school (reference)	1.00	–	–
High school	1.22	0.52–2.88	0.647
University degree or higher	1.50	0.63–3.58	0.358
**Conspiracy score**			
Low (1–4) (reference)	1.00	–	–
Middle (4–5)	0.89	0.51–1.53	0.669
High (6–7)	0.50	0.29–0.85	**0.011**
**Trust in politics and science score**			
Low (− 1.71, − 0.407) (reference)	1.00	–	–
Medium (− 0.407, 0.623)	2.92	1.66–5.13	** < 0.001**
High (0.623, 1.53)	8.76	4.80–15.97	** < 0.001**
**COVID-19 perceived risk score**			
Low (− 1.82, − 0.431) (reference)	1.00	–	–
Medium (− 0.431, 0.545)	4.85	2.79–8.45	** < 0.001**
High (− 0.545, 1.71)	13.88	7.94–24.27	** < 0.001**

Significance values are given in bold.

Belief in conspiracy theories was influenced by a limited number of factors (Supplementary Table S6): males reported a lower score than females (OR 0.61; 95% CI 0.42–0.88) and a progressive protective effect was estimated by trust in politics and science. Conversely, vaccine doubts reported a progressive and strong positive effect on the belief in conspiracy theories, in particular among those in the highest category of vaccine doubts (OR 4.74; 95% CI 2.70–8.30).

## Discussion

In this study, we investigated factors influencing the willingness to accept pharmacological (i.e., COVID-19 vaccine) and non-pharmacological (i.e., CTA Immuni) preventive measures. While previous studies independently investigated vaccine-related or digital-related acceptance^16–18,40,41^ to the best of our knowledge, this is the first article to jointly investigate which factors influenced the acceptance of both, neither or only one of the two preventive measures.

To investigate variables influencing any concordance between the COVID-19 vaccine and CTA Immuni acceptance, we used a Bayesian Bivariate Gaussian regression model. Results revealed both shared and specific factors influencing acceptance of the two preventive measures. In particular, general doubts about vaccinations, COVID-19 related risk perception, and trust in politics and science are shared factors. As for specific factors, having had the flu vaccine done in the season 2019–2020 and high education solely influenced the intention to get vaccinated, whereas job category provide a multifaceted picture, with business owner respondents less likely to accept the vaccine, and unemployed and retired ones more likely to download the app.

Hypothesis H1.a has been confirmed, as doubts about vaccinations reduced willingness to accept the COVID-19 vaccine. This finding is in line with previous literature showing that having doubts about vaccinations, in general, is a key determinant of vaccine hesitancy, both about vaccination in general^42^ and about COVID-19 vaccination in particular^25,27^. Interestingly, doubts about vaccinations in general also reduced the intention to download the Immuni CTA. Since we do not have any suggestions from the previous literature about the role of vaccine doubts on adherence to digital preventive behavior, we presume that it might be associated with other characteristics as, for example, beliefs in conspiracy theory. In support of this argument, results showed that doubts about vaccinations positively influenced beliefs in conspiracy, and negatively correlated with self-efficacy. Conspiracy and self-efficacy were not included as predictors in the model considered, since their effect was already explained by other covariates, but a possible interpretation is that both preventive behaviors might be affected by a common general source of hesitation. In particular, for the belief in conspiracy theories we showed how it was influenced by a limited set of characteristics (gender, trust in politics and science, and vaccine doubts) already considered in the main regression model. It is hoped that further studies will contribute to a better understanding of the effect. Notwithstanding, results showed that having received a flu vaccine in 2019–2020 only increased the willingness of getting vaccinated against COVID-19, in line with previous literature^25,43^ and our hypothesis H1.b. Adherence to influenza vaccination in Italy was generally very low, with an adherence of 16.8% in 2019–2020, growing to just 23.7% in 2020–2021^44^. Considering these results, it seems very important to continue to study the factors that predict adherence to vaccination, both pandemic and influenza, seeking to better plan specific and targeted intervention.

In line with our hypothesis H2.a, the perception of risk associated with COVID-19 promotes acceptance of both measures, however perceiving high risk, compared to low risk, slightly increases the discordance between the two measures. Consistently with previous studies, results showed that a high perception of risk related to COVID-19 predicts the intention to vaccinate against it^25,45^ and, to a much greater extent, to accept CTAs^17,18,21,22^. These result are in line with different models, such as the Risk as Feeling Model, according to which feelings and emotions about a specific situation influence people’s behavior who attach an emotional value to a specific situation^46^, or the Protection Motivation Theory, according to which people are motivated to respond to health threats engaging in protective behaviors^47,48^. As previously mentioned, COVID-19 related fear, worry, and uncertainty have been found to enhance the adoption of CTAs as measures to help control the spread of the disease^17,18,21,22^. Thus, a possible interpretation of our results is that perceived risk associated with COVID-19 prompts people to protect themselves against the SARS-CoV-2 infection using both vaccination and tracing apps.

In support of our hypothesis H2.b, our data showed that trust in politics and science increased the intention of getting vaccinated against COVID-19 and to download the CTA Immuni, showing that with high trust in politics and science, intention to download the CTA Immuni increased more than intention to vaccinate against COVID-19. Considering the lower motivation that participants placed on CTA Immuni compared with the vaccine, data showed that high trust in politics and science was the main determinant of the intention to download the CTA Immuni. This finding is in line with literature on CTAs, as trust in the state appeared to be, among others, a factor positively associated with the willingness to download a CTA, trust that refers to the government’s ability to ensure people’s privacy and manage access to data^17,21,49^. In addition, considering the intention to get vaccinated, previous studies showed that vaccine acceptance is positively affected by trust in the healthcare system, government, and public health researchers^32^.

Moreover, our data showed a threefold greater intention to obtain the COVID-19 vaccine than to download the Immuni CTA, and that people are more motivated to adhere to protective measures when it involves protecting others rather than protecting themselves, thus supporting our hypothesis (H3). This latter finding is in line with literature showing that the social role of the vaccine increases vaccine acceptance^29,50^, but it adds practical information on the social role that CTAs can play. CTA Immuni, like other CTAs, could have been an important weapon to counteract the COVID-19 spreading, if acceptance had been stronger. Despite intentions to download the app for the benefit of others, and the ad hoc communication promoted nationwide, people’s acceptance was low^14,15^. Understanding the reasons for such a low acceptance is a necessary precondition for the management of the current pandemic, and also in a likely scenario of future health emergencies where a timely and wide use of CTAs will allow to plan an appropriate containment.

Finally, we would like to discuss how willingness to get vaccinated and to download CTA Immuni were affected by some demographic variables that have been considered in the model as confounding factors. First, adjusting for other covariates, we found that vaccine acceptance, but not downloading the app, was more likely for higher educated people than for people with lower education. This result is in line with the literature on CTAs, which suggests that education level does not influence the intention to use or download a CTA^9^, and also with some results showing that a lower education level is associated with a higher vaccine hesitancy^51,52^. Additionally, our results show a reduced intention to get a COVID-19 vaccine and to download CTA Immuni for business owner responders, compared to employees, although only the former is significant. Previous studies^53–55^ support the finding that business owners showed more vaccine hesitancy than employees, but do not offer any possible explanation. One possible interpretation of our results is related to the fact that business owners have lower confidence in politics and science and lower perceptions of risk (Supplementary Table S7) than other participants do. Given that trust worsened during the pandemic, as governments imposed closures that particularly affected the business owner workers, it would therefore be important in future studies to understand the relationship between distrust in institutions and low risk perceptions. Conversely, retired or unemployed participants reported a greater intention to adhere to the digital tracking system. Due to the low number of participants in this job category, generalizability of this result is limited, but it still could provide insights for how communication can be targeted to strengthen CTAs acceptance.

The methodology applied to this paper, which simultaneously considers factors that predict one or more behaviors, is particularly interesting and could have important applications. Indeed, future studies should apply it to other areas, such as prevention or treatment interventions where there is a combined intervention of pharmacological and nonpharmacological therapies. Moreover, considering that this study involves only Italy, it would be particularly interesting to extend the results in a cross-cultural study, which takes into account not only the different pervasiveness of COVID-19 in different countries, but also the role of the other predictors, as for example previous vaccine behaviour and trust in politics and science.

The proposed study is not without limitations. In fact, the generalization of the evidence obtained by convenience samples remains unclear, as the derived estimates are often biased^56^. Therefore, we suggest a cautious generalization since the sample was not representative and some combinations of categories among variables reported a low sample size. Although results were adjusted for a standard set of variables (age, gender and socio-demographic characteristics) as an attempt to improve generalizability, future studies based on probability samples are needed to corroborate the reported findings. As a further limitation, the study investigated only one pharmacological measure, the COVID-19 vaccines, and one non-pharmacological measure, the CTA Immuni, as preventive measures. This choice stemmed from the fact that these two measures, more than others, such as social distancing and wearing a mask, are often associated with inherent fears (e.g., side effects and invasion of privacy) that strongly reduce acceptance.

## Conclusion

It is widely believed that the pandemic will end thanks to different factors: maintaining a high pace of vaccine production and distribution, promoting vaccine acceptance, and supporting acceptance of practices such as social distancing and wearing masks, in addition to tracing apps. Our results further highlight that adherence to protective behaviors is predicted by both similar and specific factors. An effective communication should consider not only aspects closely related to the pandemic, such as risk perception, or specific behaviors related to protective measures, such as trust in institutions and conspiracy theories, but also lean on the motivation (i.e. the protection of others) of why people choose to adhere to such protective measures (COVID-19 vaccine and CTA Immuni). A conscious communication effort, focusing on these peculiarities, could encourage greater acceptance of the behaviors examined, allowing greater control and containment of the spread of the virus.

## Supplementary Information

Supplementary Information.
