# Supplementary material for: Joint analysis of the intention to vaccinate and to use contact tracing app during the COVID-19 pandemic
Source: Sci Rep. 2022 Jan 17;12:793. doi: 10.1038/s41598-021-04765-9 (PMC8764077; doi:10.1038/s41598-021-04765-9)

**Title: Joint analysis of the intention to vaccinate and to use contact tracing app during the COVID-19 pandemic.**

Caserotti Marta^1^, Girardi Paolo^1,2^*, Tasso Alessandra^3^, Rubaltelli Enrico^1^,

Lotto Lorella^1^, Gavaruzzi Teresa^1^

**Supplementary material**

**Supplementary Note**

**Questionnaire**

1. **If a Coronavirus vaccine were available in the upcoming fall season, would you vaccinate?**

Please, answer by moving the slider to indicate your degree of agreement with the question. The value 0 corresponds to *Absolutely unlikely*, and 100 corresponds to *Absolutely likely*.

1. **Do you plan to use the Immuni app for positive case tracking?**

Answer by moving the slider to indicate your degree of agreement with the question. The value 0 corresponds to *Absolutely no*, and 100 corresponds to *Absolutely yes*.

1. Measures taken to combat Coronavirus, as is generally the case with diseases caused by infectious agents, have two goals: 1) to protect the health of the individual who implements these protective measures; 2) to protect others and in particular those who are most at risk (e.g., people over the age of 65 and/or with medical conditions).
   - I would use the Immuni application to: (Please, answer by indicating a value from 1 to 4, where 1 corresponds to *Not at all* and 4 to *Very much*).
     - Protect myself/ Protect others
     - Protect others/ Protect myself
   - I would vaccinate for Coronavirus, if it were available, to: (Please, answer by indicating a value from 1 to 4, where 1 corresponds to *Not at all* and 4 to *Very much*).
     - Protect myself/ Protect others
     - Protect others/ Protect myself
2. On a scale of 0 to 100, **how scared do you feel of the Coronavirus**?

Please, answer by moving the slider to indicate your degree of agreement with the question. The value 0 corresponds to *Not at all scared*, while the value 100 corresponds to *Extremely scared*.

1. On a scale of 0 to 100, **how severe do you think the illness caused by Coronavirus is**?

Please, answer by moving the slider to indicate your degree of agreement with the question. The value 0 corresponds to *Not at all serious*, while the value 100 corresponds to *Extremely serious*.

1. On a scale of 0 to 100, **how likely do you think you are to get sick from the Coronavirus**?

Please, answer by moving the slider to indicate your degree of agreement with the question. A value of 0 corresponds to *Not at all likely*, while a value of 100 corresponds to *Extremely likely*.

1. On a scale of 0 to 100 (where 0 corresponds to *Not at all*, and 100 corresponds to *Extremely*), **how dubious do you feel about vaccines**?
2. **How much do you think your behaviours can help solve the Coronavirus-related health emergency**?

Please, answer by moving the slider to indicate your level of agreement with the question. The value 0 corresponds to *Not at all*, and 100 corresponds to *Absolutely*.

1. At this moment, **how much trust do you have in the institutions**?

Please, answer by moving the slider to indicate your level of agreement with the question. The value 0 corresponds to *Not at all trustful*, and 100 corresponds to *Absolutely trustful*.

o International institutions (e.g. World Health Organization)

o National institutions (e.g. Government)

o Regional institutions (e.g. Regions)

o Scientific committees (e.g. Task Force)

1. Have you personally **become ill from the Coronavirus**? Yes/No
2. Do you directly **know someone who has become ill from the Coronavirus**? Yes/No
3. Some political and social events are being questioned (e.g. the 9/11 attacks, the death of Lady Diana, the assassination of John F. Kennedy). It is suggested that the "official version" of these events may be an attempt to hide the truth from the public. This "official version" could mask the fact that these events were secretly planned and prepared for by a secret alliance of powerful individuals or organizations (e.g., intelligence or government).

What do you think? To answer, please indicate the extent to which the sentence below represents your views: **I think that the "official version" of events that is provided by the authorities very often hides the truth**.

Please respond by indicating a value from 1 to 7, where 1 corresponds to *Completely False* and 7 to *Completely True*.

1. Gender:
   - Male
   - Female
2. Age (integer number, years old)
3. Income
   - <10.000
   - 10.001/15.000
   - 15.001/26.000
   - 26.001/55.000
   - 55.001/75.000
   - 75.001/120.000
   - more than 120.000
   - I prefer not to answer
4. Please indicate the highest level of schooling you have attained:
   - Middle school diploma
   - High school diploma
   - University degree or higher
5. Please indicate the municipality in which you live.
6. Please also include the ZIP code of the municipality where you live.
7. Please state your occupation:
   - Employee
   - Business owner
   - Retired - Unemployed
   - Student

Questions 1, 4, 5, 6, and 7 are the same as Caserotti et al., 2021. Question 2 was adapted from Walrave et al., 2021. Questions 3 and 8 were developed ad hoc to investigate the research questions. Question 9 was adapted from Lewandowsky, S. *et al*. 2021 and Kostka et al., 2020. Question 12 was adapted by Lantian et al., 2016.

**Table S1.** Selection Criteria. Presence of informed consent, questionnaire completion of at least 80% and completeness of the analysed variables.

| **Selection Criteria*** | **Total**  **(*n* = 668)** |
| --- | --- |
| Informed consent, n (%) |  |
| Yes | 662 (99.1) |
| No | 6 (0.9) |
| Questionnaire completion ≥ 80%, n (%) |  |
| Yes | 476 (71.3) |
| No | 192 (28.7) |
| Presence of missing values on the analysed variables, n (%) |  |
| Yes | 214 (32.0) |
| No | 454 (68.0) |
| Questionnaires included in the analysis, n (%) |  |
| Yes | 448 (67.1) |
| No | 220 (32.9) |

**selection criteria are not mutually exclusive.*

**Table S2.** Loadings of the factorial analysis for Trust in politics and science score and COVID-19 perceived risk score.

| **Trust in politics and science score** | | **COVID-19 perceived risk score** | |
| --- | --- | --- | --- |
| Trust international institutions  (Median: 60; IQR: 30-81) | 0.838 | Scare  (Median: 50 IQR: 29-73) | 0.954 |
| Trust national institutions  (Median: 48; 14-70) | 0.794 | Severity  (Median 70; IQR 49-85) | 0.762 |
| Trust scientific committees  (Median: 62; IQR:30-85) | 0.899 | Likelihood of being infected  (Median 70; IQR: 50 28-54) | 0.558 |
| Var. % explained | 71.4% | Var. % explained | 60.0% |

**Table S3.** DIC Value for variable model selection. In bold the minimum value.

| **Model** | **DIC** |
| --- | --- |
| Starting model* | 8477 |
| + Educational level | 8466 |
| + Educational level, Job category | **8459** |
| + Educational level, Job category, Salary | 8478 |
| + Educational level, Job category, Trust in local institution | 8471 |
| + Educational level, Job category, Conspiracy | 8474 |
| + Educational level, Job category, Self-efficacy | 8465 |
| + Educational level, Job category, Direct COVID-19 contact | 8464 |

**Included covariates: Vaccine doubts index category, Flu vaccine 2019-2020 done, COVID-19 perceived risk score category, Age with 5 basis of cubic splines, Trust in politics and science score category and gender.*

**Table S4.** Motivation score to take a measure against COVID-19 by measure motivation (for other, for myself) and measure type (CTA Immuni, COVID-19 vaccine) by terciles of the COVID-19 perceived risk score.

|  |  | **COVID-19 perceived risk score** | | |  |
| --- | --- | --- | --- | --- | --- |
| **Motivation score** | **Overall**,  N = 448*^1^* | **[-1.82,-0.431]**,  N = 149*^1^* | **(-0.43,0.545]**,  N = 149*^1^* | **(0.545,1.71]**,  N = 150*^1^* | **P-value***^2^* |
| **CTA Immuni for others** | 3 (2, 4) | 2 (1, 3) | 3 (2, 4) | 4 (3, 4) | <0.001 |
| **CTA Immuni for myself** | 3 (2, 4) | 2 (1, 3) | 3 (2, 4) | 4 (2, 4) | <0.001 |
| **COVID-19 vaccine for others** | 4 (3, 4) | 3 (1, 4) | 4 (3, 4) | 4 (4, 4) | <0.001 |
| **COVID-19 vaccine for myself** | 4 (2, 4) | 2 (1, 4) | 4 (3, 4) | 4 (3, 4) | <0.001 |
| *^1^*Median (IQR), *^2^*Kruskal-Wallis rank sum test by COVID-19 perceived risk score. | | | | | |

**Table S5.** Adjusted* coefficient and related 95% CI estimated by a Bayesian Bivariate Gaussian regression model for the scale parameter ($log(\sigma_{1})$ and $log(\sigma_{2})$) of the score of likelihood to get a COVID-19 vaccine and to download CTA Immuni respect to reference category**.

|  | **COVID-19 vaccine** | | | **CTA Immuni** | | | | |
| --- | --- | --- | --- | --- | --- | --- | --- | --- |
| **Estimates** | $\hat{\gamma}_{1}$ | **95% CrI** | | $\hat{\gamma}_{2}$ | **95% CrI** | | |  |
| **(Intercept)** | 3.81 | 3.47 | 4.17 | 3.71 | 3.33 | 4.11 |  |  |
| **Vaccine doubts index** |  |  |  |  |  |  |  |  |
| Low [1,14] | -0.13 | -0.35 | 0.10 | 0.04 | -0.18 | 0.26 |  |  |
| Medium (14,50] | **0.21** | **0.00** | **0.43** | -0.07 | -0.28 | 0.14 |  |  |
| High (50,100] | **0.28** | **0.04** | **0.50** | -0.13 | -0.37 | 0.15 |  |  |
| **Flu vaccine 2019-2020 done** [Yes] | **-0.66** | **-0.93** | **-0.36** | 0.06 | -0.17 | 0.29 |  |  |
| **COVID-19 perceived risk score** |  |  |  |  |  |  |  |  |
| Medium (-0.431,0.545] | -0.19 | -0.36 | 0.00 | -0.07 | -0.26 | 0.12 |  |  |
| High (-0.545,1.71] | -0.17 | -0.38 | 0.06 | 0.07 | -0.13 | 0.26 |  |  |
| **Trust in politics and science score** |  |  |  |  |  |  |  |  |
| Medium (-0.407,0.623] | **-0.24** | **-0.42** | **-0.06** | **0.30** | **0.12** | **0.48** |  |  |
| High (0.623,1.53] | **-0.35** | **-0.55** | **-0.11** | **0.21** | **0.01** | **0.39** |  |  |
| **Gender** [Male] | -0.03 | -0.20 | 0.15 | -0.04 | -0.22 | 0.13 |  |  |
| **Job** |  |  |  |  |  |  |  |  |
| Business-owner | -0.24 | -0.46 | 0.00 | -0.14 | -0.38 | 0.11 |  |  |
| Retired-Unemployed | -0.11 | -0.42 | 0.23 | -0.18 | -0.46 | 0.12 |  |  |
| Student | -0.03 | -0.27 | 0.21 | -0.12 | -0.34 | 0.09 |  |  |
| **Education level** |  |  |  |  |  |  |  |  |
| High school | **-0.41** | **-0.73** | **-0.12** | -0.25 | -0.59 | 0.06 |  |  |
| Degree or higher | **-0.44** | **-0.80** | **-0.12** | -0.21 | -0.55 | 0.13 |  |  |

**Adjusted also by age with penalized cubic splines with 5 equally spaced knots (Figures S2).*

*** Reference category: Vaccine doubts index [No doubt], Flu vaccine 2019-2020 done [No], COVID-19 perceived risk score [Low], Trust in politics and science score [Low], Gender [Female], Job [Employee], Education level [Middle school].*

**Table S6.** Adjusted* OR and related 95% CI estimated by a Cumulative Logistic regression model for the belief on conspiracy theories.

| **Characteristics** | **OR** | **95% CI** | **P-value** |
| --- | --- | --- | --- |
| **Vaccine doubts index** |  |  |  |
| Low [1,14] | 1.20 | 0.73 – 1.98 | 0.470 |
| Medium (14,50] | 1.64 | 1.01 – 2.68 | 0.047 |
| High (50,100] | 4.74 | 2.70 – 8.30 | <0.001 |
| **Trust in politics and science score** |  |  |  |
| Medium (-0.407,0.623] | 0.46 | 0.30 – 0.70 | <0.001 |
| High (0.623,1.53] | 0.28 | 0.17 – 0.44 | <0.001 |
| **Gender** [Male] | 0.61 | 0.42 – 0.88 | 0.008 |

**Reference category: Vaccine doubts index [No doubts], Trust in politics and science score [Low], Gender [Female].*

**Table S7.** Main characteristics of the participants by job category. Tests are performed between characteristics and job category.

| **Characteristics** | **Job category** | | | | **P-value***^1^* |
| --- | --- | --- | --- | --- | --- |
|  | **Employee**,  N = 186 | **Business-owner**,  N = 53 | **Retired-Unemployed**,  N = 35 | **Student**,  N = 174 |  |
| **Gender** (Females), N (%) | 124 (67%) | 31 (58%) | 29 (83%) | 133 (76%) | **0.014** |
| **Age**, Median (IQR) | 38 (27, 52) | 45 (34, 52) | 51 (33, 64) | 22 (20, 24) | **<0.001** |
| **Educational level**, N (%) |  |  |  |  | **0.002** |
| Middle school | 14 (7.5%) | 6 (11%) | 8 (23%) | 5 (2.9%) |  |
| High school | 77 (41%) | 22 (42%) | 17 (49%) | 81 (47%) |  |
| Degree or higher | 95 (51%) | 25 (47%) | 10 (29%) | 88 (51%) |  |
| **Family status**, N (%) |  |  |  |  | **<0.001** |
| Single | 61 (33%) | 19 (36%) | 7 (20%) | 133 (76%) |  |
| Married – Living Together | 110 (59%) | 32 (60%) | 25 (71%) | 19 (11%) |  |
| Others | 15 (8.1%) | 2 (3.8%) | 3 (8.6%) | 22 (13%) |  |
| **Salary,** N (%) |  |  |  |  | **<0.001** |
| <15k | 48 (26%) | 13 (25%) | 15 (43%) | 47 (27%) |  |
| 15-55k | 113 (61%) | 20 (38%) | 13 (37%) | 53 (30%) |  |
| >55k | 11 (5.9%) | 15 (28%) | 1 (2.9%) | 17 (9.8%) |  |
| Unknown | 14 (7.5%) | 5 (9.4%) | 6 (17%) | 57 (33%) |  |
| **Flu vaccine 2019-2020 done**, N (%) | 21 (11%) | 2 (3.8%) | 8 (23%) | 19 (11%) | 0.051 |
| **Likelihood to get a COVID-19 vaccine**, Median (IQR) | 81 (11, 100) | 20 (0, 52) | 90 (46, 100) | 100 (81, 100) | **<0.001** |
| **Likelihood to download CTA Immuni**, Median (IQR) | 50 (0, 86) | 0 (0, 40) | 50 (24, 96) | 54 (16, 91) | **<0.001** |
| **Trust in politics and science score**, Median (IQR) | -0.12 (-0.81, 0.73) | -1.15 (-1.60, -0.28) | 0.19 (-1.01, 0.55) | 0.59 (-0.08, 0.97) | **<0.001** |
| **Trust in local institution score**, Median (IQR) | 60 (30, 80) | 55 (30, 70) | 61 (46, 85) | 60 (35, 80) | 0.61 |
| **Self-efficacy score**, Median (IQR) | 80 (50, 96) | 70 (30, 82) | 71 (50, 86) | 80 (56, 92) | **0.019** |
| **Conspiracy score**, Median (IQR) | 4.50 (3.00, 6.00) | 5.00 (4.00, 6.00) | 4.00 (3.00, 5.00) | 4.00 (3.00, 5.00) | **0.007** |
| **COVID-19 perceived risk score**, Median (IQR) | 0.10 (-0.68, 0.91) | -0.99 (-1.61, -0.50) | 0.15 (-0.76, 0.88) | 0.18 (-0.36, 0.80) | **<0.001** |
| **Direct COVID-19 contact**, N (%) | 110 (59%) | 32 (60%) | 17 (49%) | 89 (51%) | 0.32 |

*^1^Pearson's Chi-squared test; Kruskal-Wallis rank sum test*

**Figure S1.** Marginal distribution and pairwise Pearson’s correlation between variables included in the factorial analysis for Trust in politics and science score (a) and COVID-19 perceived risk score (b). Significance test is reported*.

(a) (b)

**
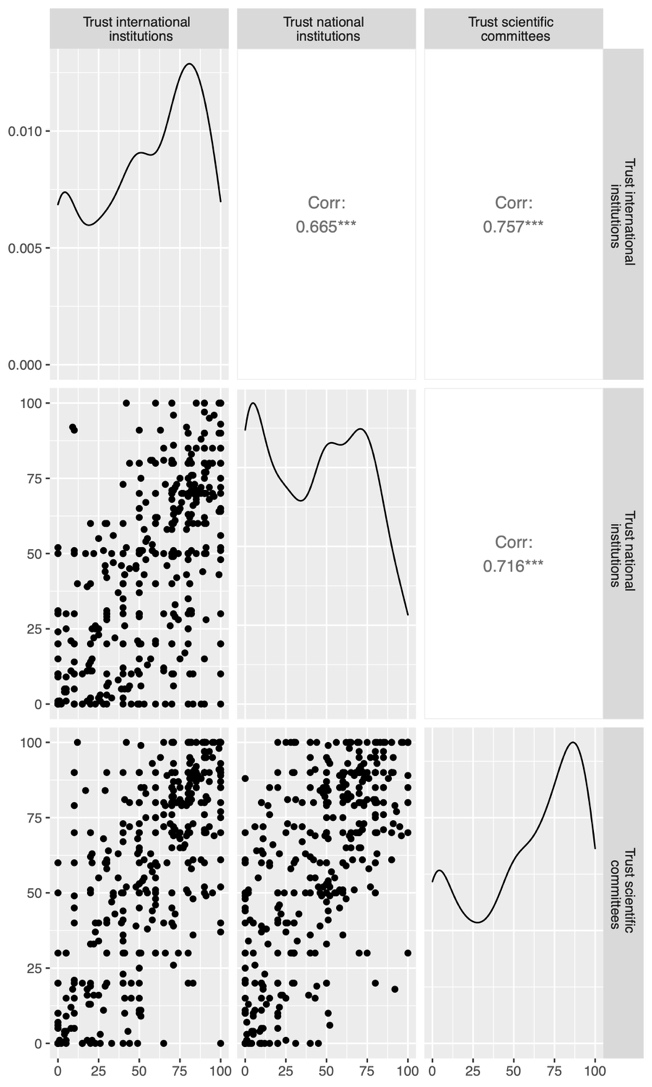

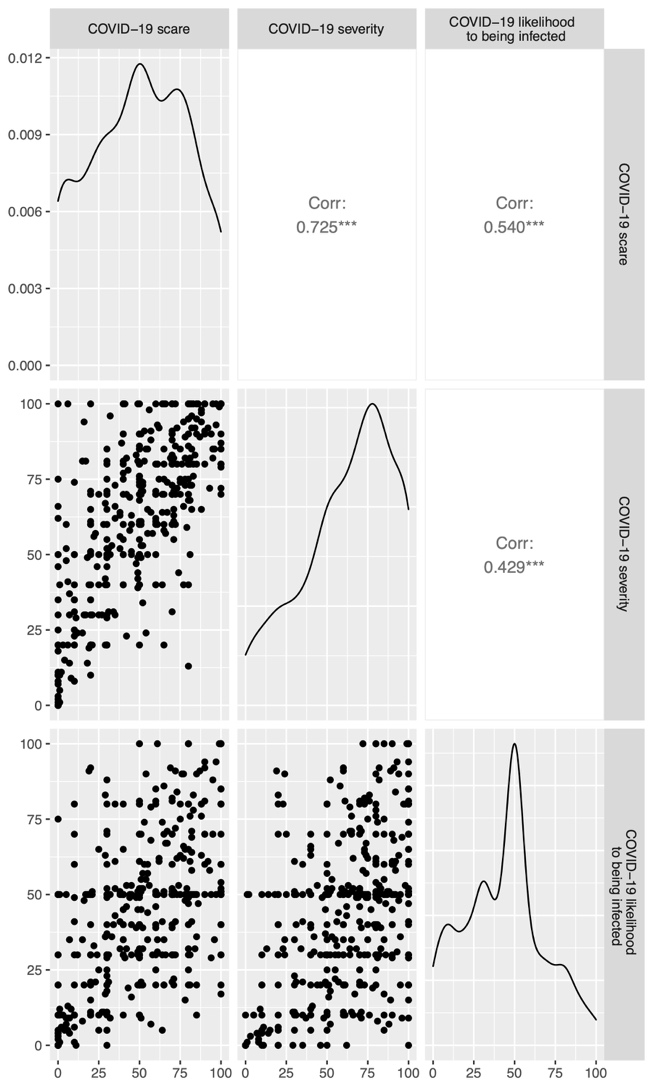
**

**Pearson’s moment correlation test: *p<0.05, **p<0.01, ***p<0.001.*

**Figure S2.** Adjusted effect of age estimated by a Bayesian Bivariate Gaussian regression model on the linear predictor for the scale parameter ($log(\sigma_{1})$ and $log(\sigma_{2})$) of the score of likelihood to get a COVID-19 vaccine (a) and to download CTA Immuni (b).

1. (b)


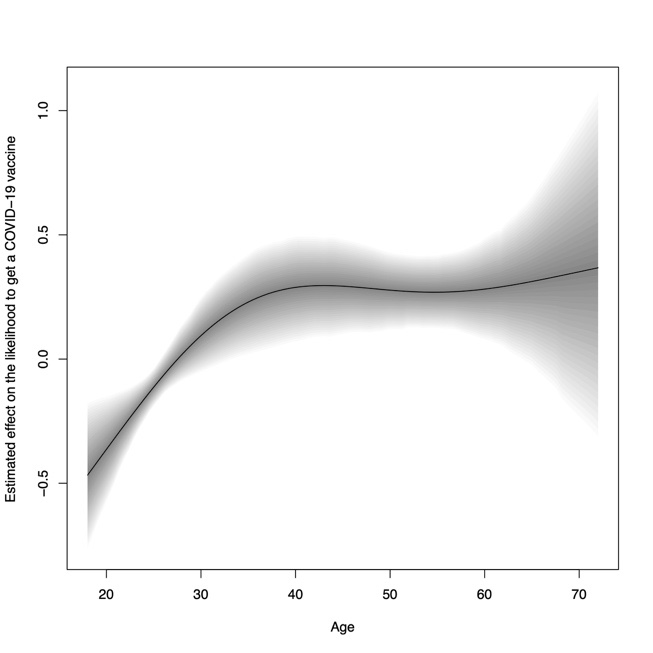

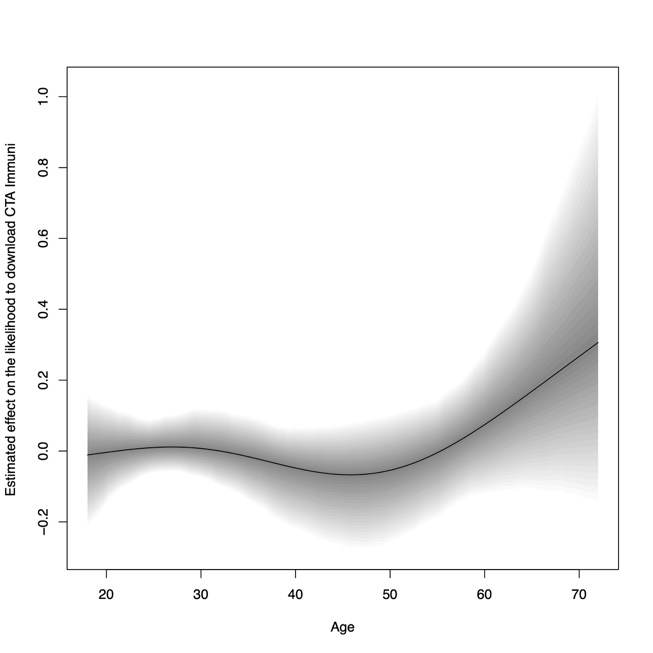


**Figure S3.** Quantile-quantile diagram for sample residuals of the estimated Bayesian Bivariate Gaussian Regression Model for the score of likelihood to get a COVID-19 vaccine (a) and to download CTA Immuni (b) compared to theoretical quantiles.

(a) (b)


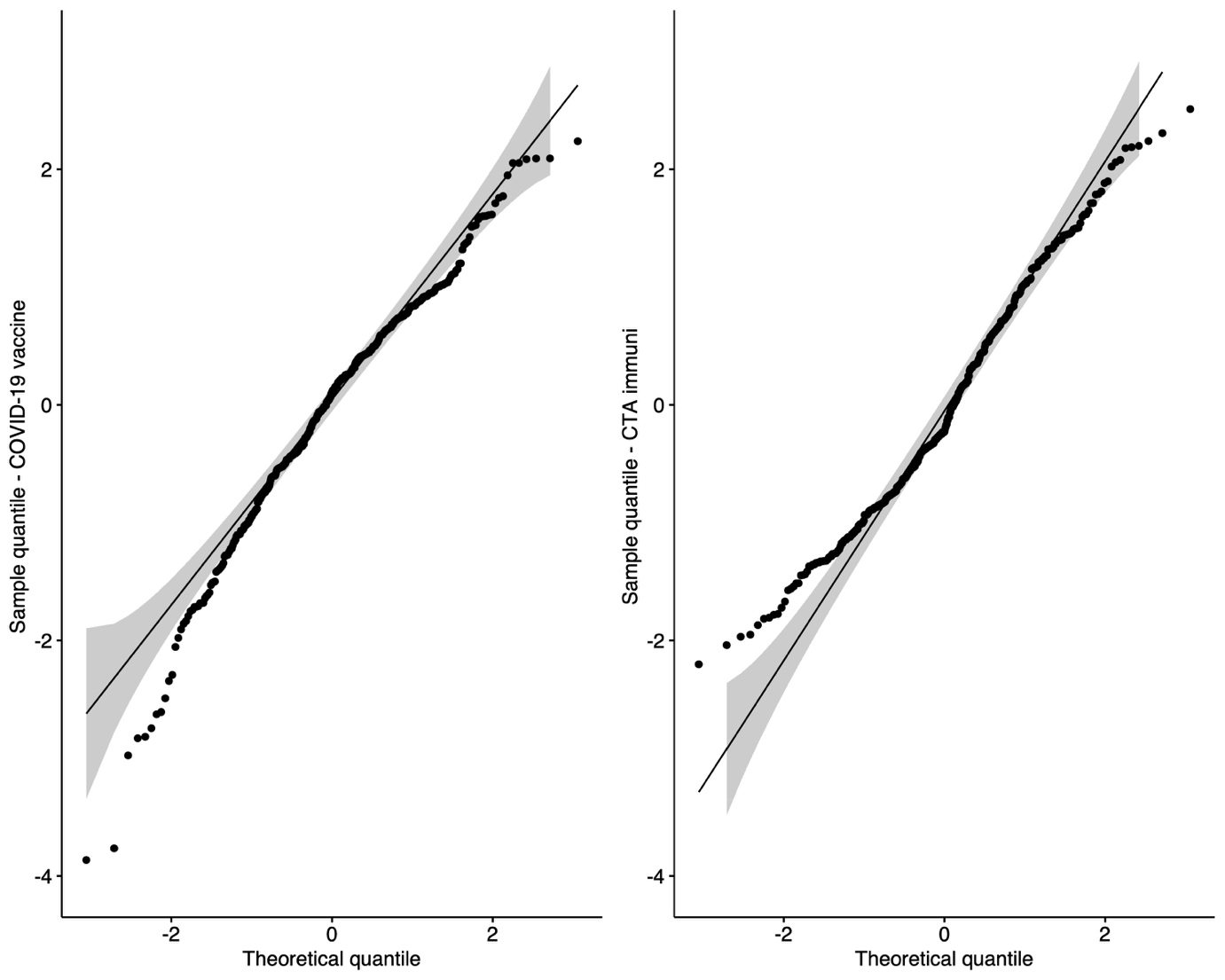

Supplement: Supplementary file 1 — Supplementary Information. [file 41598_2021_4765_MOESM1_ESM.docx]
